# Complexity traits and synchrony of cryptocurrencies price dynamics

**DOI:** 10.1007/s10203-021-00319-w

**Published:** 2021-02-16

**Authors:** Davide Provenzano, Rodolfo Baggio

**Affiliations:** 1 Department of Economics, Business and Statistics (SEAS), University of Palermo, 90128 Palermo, Italy; 2 Master in Economics and Tourism and Dondena Center for Research on Social Dynamics and Public Policy, Bocconi University, Milan, Italy; 3 School of Core Engineering Education, Tomsk Polytechnic University, Tomsk, Russian Federation

**Keywords:** Cryptocurrency, Time series, Complex network analysis, Synchronization, Bitcoin, C32, D53, C6

## Abstract

In this study, we characterized the dynamics and analyzed the degree of synchronization of the time series of daily closing prices and volumes in US$ of three cryptocurrencies, Bitcoin, Ethereum, and Litecoin, over the period September 1,2015–March 31, 2020. Time series were first mapped into a complex network by the horizontal visibility algorithm in order to revel the structure of their temporal characters and dynamics. Then, the synchrony of the time series was investigated to determine the possibility that the cryptocurrencies under study co-bubble simultaneously. Findings reveal similar complex structures for the three virtual currencies in terms of number and internal composition of communities. To the aim of our analysis, such result proves that price and volume dynamics of the cryptocurrencies were characterized by cyclical patterns of similar wavelength and amplitude over the time period considered. Yet, the value of the slope parameter associated with the exponential distributions fitted to the data suggests a higher stability and predictability for Bitcoin and Litecoin than for Ethereum. The study of synchrony between the time series investigated displayed a different degree of synchronization between the three cryptocurrencies before and after a collapse event. These results could be of interest for investors who might prefer to switch from one cryptocurrency to another to exploit the potential opportunities of profit generated by the dynamics of price and volumes in the market of virtual currencies.

## Introduction

The rapid and successful diffusion of Bitcoin and digital currencies, as a practical mean of payment for online services and goods and substitute of traditional money assets, has attracted a remarkable attention. Much of the social debate centers on cyber-security, legitimacy, and reputation issues due to possible hacking attacks of the peer-to-peer network used for the electronic payments, which allows anonymous transactions and carries risks as money laundering, the financing of criminality and terrorism, and tax evasion.

More recently, the high volatility of price series has fostered an increasing interest in bubbles detection and price dynamics of cryptocurrencies in the associated markets (Garcia et al. 2014; Bouoiyour et al. 2015; Donier and Bouchaud 2015; Hencic and Gouriéroux 2015; Blau 2017).

Bariviera (2017), Bariviera et al. (2017), and Lahmiri et al. (2018) studied the long-range dependence of return and volatility, and other statistical features of Bitcoin daily and intraday prices, from 2011 to 2017. They found the long-range correlation behavior in the daily volatility series of Bitcoin. Lahmiri and Bekiros (2018) showed that, as opposed to returns, Bitcoin prices incorporate and exhibit chaotic dynamics and nontrivial correlation patterns at different time scales. By using methods that originate in physics, Cheah and Fry provided empirical evidence that Bitcoin prices contain a substantial speculative bubble component (Cheah and Fry 2015) and identified shocks and crashes in cryptocurrency markets with specific evidence for negative bubbles (Fry and Cheah 2016). Corbet et al. (2018) related Bitcoin and Ethereum prices to ‘‘fundamental drivers’’ to reveal the existence and dates of bubbles periods in the market. By applying the methodology discussed in Phillips et al. (2015), Cheung et al (2015), Li et al. (2019), and Bouri et al. (2019) aimed at detecting a bubble behavior in price dynamics at some point in time. Chaim and Laurini (2019) and Cretarola and Figà-Talamanca (2019b) related to strict local martingale theory to investigate price dynamics of Bitcoin by a continuous time stochastic model. More precisely, Chaim and Laurini (2019) estimated the volatility function of Bitcoin daily and high-frequency five minutes prices, whereas in Cretarola and Figà-Talamanca (2019b) the association of cryptocurrencies price dynamics with investors’ attention and sentiment is described with a regime-switching correlation parameter. The impact of market attention on Bitcoin returns and volatility is also the research interest in Kristoufek (2013, 2015), Figà-Talamanca and Patacca (2019), Cretarola et al. (2019), Cretarola and Figà-Talamanca (2019a). Lahmiri and Bekiros (2020) explored the evolution of the informational efficiency in 45 cryptocurrency markets and 16 international stock markets before and during COVID-19 pandemic. They found that investing in digital assets during big crises could be considered riskier as opposed to equities, as cryptos showed more instability and more irregularity during the COVID-19 pandemic compared to international stock markets.

For the study of dynamical systems, Luque et al. (2011) have proposed a novel method, called the horizontal visibility graph (HVG) algorithm, which captures the nature of different classes of series in a network context. By the HVG algorithm, time series are proficiently converted into a network representation and then analyzed from a new and complementary viewpoint, and with a full set of alternative techniques and tools from the complex network theory. Most of all, the topology of the network inherits the structure of the time series, in such a way that periodic, random, and fractal series map into motif-like, random exponential and scale-free networks, respectively (Bollobás 1998; Watts and Strogatz 1998; Barabási and Albert 1999).

However, in spite of its suitability for the study of time series, the application of HVG algorithm to cryptocurrencies price dynamics is limited to the study carried out in Liu et al. (2020) to investigate the Bitcoin price volatility and deepen the understanding of the markets for rare items, e.g., the gold market.

In this paper, we took advantage of the HVG algorithm to derive information about the process that generates the time series of three cryptocurrencies, Bitcoin (BTC), Ethereum (ETH), and Litecoin (LTC), over the period September 1, 2015–March 31, 2020.

Actually, BTC, ETH, and LTC are the most popular, big name cryptocurrencies. As of August 2020, BTC and ETH are the digital coins with the largest market capitalization (assets in circulation multiplied by asset price) just below 218 and 46 billion of US dollars (Investing.com 2020), respectively. LTC stays behind Bitcoin as the seventh largest digital currency by market cap. It is often referred to as “silver to Bitcoin’s gold” as LTC adopts many of the features of BTC, and changes some other aspects that in 2011 the founder Charlie Lee felt could be improved. In fact, compared to BTC, LTC can produce a greater number of coins and it is characterized by faster transactions, lower transaction fees, and a new cryptographic algorithm for a more easily accessible process, said “mining,” for generating and releasing new coins and for verifying, authenticating, and then adding the ongoing network transactions to a public ledger.

Among the three cryptocurrencies considered, ETH is the youngest one as it was launched by Vitalik Buterin on July 30, 2015. Therefore, we chose to consider a time frame starting from September 1, 2015, in order to have three time series of the same length.

Besides price and volume dynamics, in the proposed study, we also investigated the degree of synchronization between the three leading cryptocurrencies cited to detect potential interactions among bubble periods within the cryptocurrency markets.

Hence, two research questions guided our analysis. As a first concern, we questioned whether the source of unpredictability in the price dynamics of the virtual currencies under study origins in a chaotic, deterministic, or stochastic dynamical system, which is a fundamental issue for modeling and forecasting purposes. To this end, we used results from Lacasa and Toral (2010) showing that the three series map into a graph with exponential degree distribution, $P\left( k \right)\sim \exp \left( { - \lambda k} \right)$, where the value of λ characterizes the specific process that generated the series. Second, we investigated whether price and volume upswings and crushes in one cryptocurrency market can lead to similar dynamics in other cryptocurrencies by running a synchrony analysis for the series investigated. To this aim, we followed the procedure described in Freeman et al. (2018) and Cazelles (2004). Potential interactions among bubble periods within the cryptocurrency market might influence diversification possibilities and trading strategies. In fact, an investors might prefer to switch from one cryptocurrency to another to exploit the opportunities of profit generated by the correlated behavior of virtual currencies. The above research questions represent where our paper seeks to contribute to the existing literature.

The remainder of the paper is structured as follows. We start in Sect. 2 with the description of the data and methods set used in our study. In Sect. 3, we focus on the empirical analysis and summarize our results. Section 4 concludes.

## Materials and methods

Daily closing prices and volumes in US$ of three virtual currencies, Bitcoin (BTC), Ethereum (ETH), and Litecoin (LTC) were downloaded from the Web site https://uk.investing.com (2020), for a total of 1680 trading days over the period September 1, 2015–March 31, 2020. The research period resulted bounded by the shorter dataset available for Ethereum, given the necessity to overlap the three series.

**Fig. 1:**
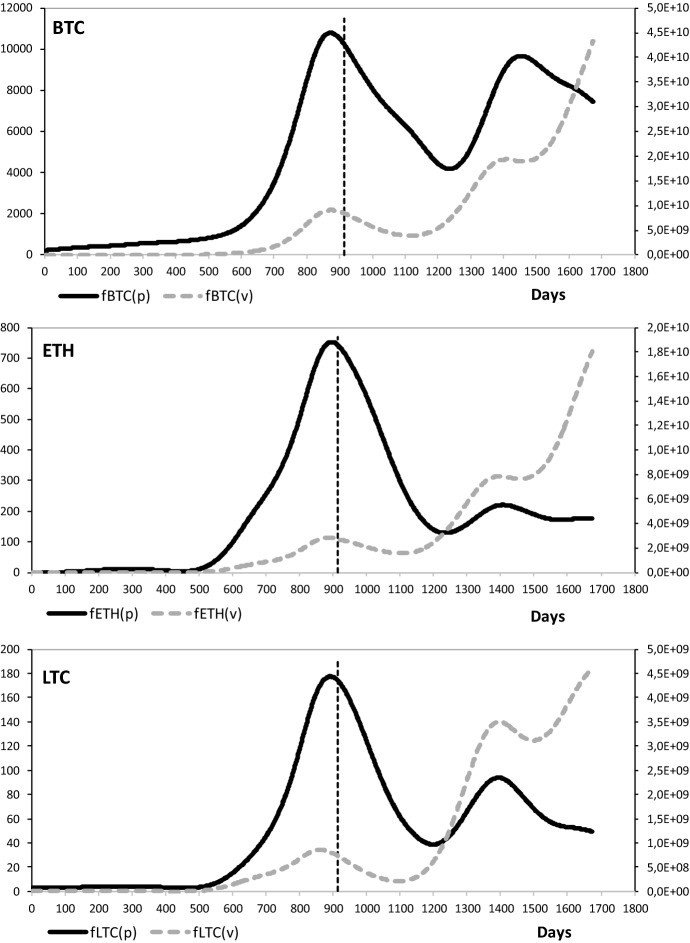
Daily prices (p) and volumes (v) series in US$ of Bitcoin (BTC), Ether (ETH), and Litecoin (LTC), smoothed with the Hodrick–Prescott filter [*μ* = 6,250,000 (Baggio and Klobas 2017)]. The black solid curve represents prices whereas the gray dotted curve represents exchange volumes of the cryptocurrency

As shown in Fig. 1, raw data were first Hodrick–Prescott (HP, Hodrick and Prescott 1997) filtered to remove short-term fluctuations associated with the business cycles and reveal long-term trends.

The HP filter is a nonparametric, nonlinear optimization algorithm used to remove the cyclical component (short-term fluctuations) of a time series from raw data. Basically, the series is divided into its growth (long-term trend) and cyclical components so that the squared deviation of the values from the trend is minimized. The HP filter is controlled by a smoothness parameter *μ*, which penalizes variability in the growth component series. The larger the value of *μ*, the higher the penalty, the smoother the long-term component (as *μ* approaches infinity the filter produces a line; *μ* = 0 leaves unmodified the series). Literature suggests optimal values for *μ* depending on the frequency of observations. Here we used *μ* = 6,250,000, which is the value recommended for daily data (Baggio and Klobas 2017).

Then, the horizontal visibility (HV) algorithm was used to map the smoothed time series into graphs according to the specific geometric criterion described in Lacasa et al. (2008).

In fact, in order to analyze the complex features of the dynamics shown by the cryptocurrencies investigated, we avoided methods as the Lyapunov exponents, the Hurst exponent, fractal dimensions, symbolic discretization, and measures of complexity such as entropies or quantities derived from them (Kantz and Schreiber 2003; Sprott 2003), whose calculation requires sophisticated techniques and the interpretation of results can be problematic for practitioners with little expertise (Baggio 2008; Baggio and Sainaghi 2011).

Instead, the HVG approach is characterized by a straightforward implementation, is computationally less complex than the cited methods, and provides us with a quite simple mapping method for inheriting the time series properties in the structure of the associated graphs. “These features are going to make it easier to find connections between the underlying processes and the networks obtained from them by a direct analysis of the latter” (Núñez et al. 2012, p. 121).

Let $Y = \left\{ {y_{i} } \right\}_{i = 1,2, \ldots , n}$ be a time series with *n* observations. Each data point $y_{i}$ in the series is regarded as a node in the associated network graph, and hence, nodes inherit a natural ordering. For any two arbitrary nodes, *m* and *n*, they are said to have “horizontal visibility” to each other, and hence an edge connects them in the associated graph, if any other node *h* between them is associated with a lower record $y_{h}$ in the series. Formally, a horizontal visibility edge exists between two nodes *m* and *n*, if $y_{m} > y_{h}$ and $y_{n} > y_{h}$, for every node *h* such that $m < h < n$.

The network extracted from a time series with the described algorithm is by construction always undirected connected, as any data point in the series sees at least its nearest neighbors (Li et al. 2012).

**Fig. 2:**
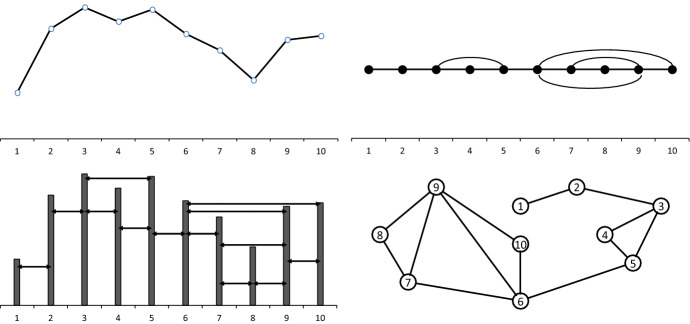
Representation of the algorithm to transform the last 10 data points in the Ethereum time series into the associated HVG

For illustrative purposes, the horizontal visibility graph (HVG) algorithm is represented in Fig. 2, where vertical bars are used to plot into the corresponding graph the latest 10 data points in the Ethereum time series.

The main properties of the HVG representation can be found in Luque et al. (2009). Here we just recall that a time series mapped into an HVG with an exponential degree distribution, $P\left( k \right)\sim \exp \left( { - \lambda k} \right)$, shows chaotic, uncorrelated, or correlated stochastic dynamics depending on the value of the slope $\lambda$. In particular, in Luque et al. (2009), the critical value $\lambda_{{\text{c}}} = {\text{ln}}\left( {3/2} \right)$ of the exponent has been found for the case of uncorrelated noise (white noise), by analytically computing the degree distribution of the HVG associated with a bi-infinite sequence of independent and identically distributed random variables extracted from a continuous probability density function.

Thus, chaotic series map into an HVG with $\lambda < {\text{ln}}\left( {3/2} \right)$, the slope is exactly on the frontier $\lambda_{{\text{c}}} = {\text{ln}}\left( {3/2} \right)$ for an uncorrelated random series, and $\lambda > {\text{ln}}\left( {3/2} \right)$ characterizes a correlated stochastic process (Lacasa and Toral 2010). Therefore, the higher the slope of the exponential degree distribution, the higher the stability and predictability of the system.

The existence of significant fluctuation cycles in the time series under study was also investigated looking at the community structure shown by the associated networks. Networks often show a structure organized in communities (or modules), where nodes belonging to a community are more densely connected among them than with nodes outside the group.

Communities are loosely connected to each other instead (Newman and Girvan 2004; Fortunato 2010). The extent to which a network can be divided into well recognizable communities is measured by the index of modularity

$$ Q = \mathop \sum \limits_{i} \left( {e_{ii} - a_{i} } \right)^{2} , $$

where *e*_*ii*_ represents the fraction of connections between nodes belonging to the same module *i* and *a*_*i*_ is the fraction of links with at least one end node inside module *i*. *Q* is normalized between 0 (absence of modules) and 1 (perfect division into completely separated groups).

Here we used the algorithm described in Bondel et al. (2008) to identify the different modules and derive the value of *Q*. Basically, the technique implements an iterative model to determine the optimal number of partitions that maximize the index *Q*, given a resolution parameter to determine the granularity level at which communities are detected. In our analysis, we set the resolution equal to 1 to get a standard modularity-based community detection.

Finally, the method proposed in Freeman et al. (2018) and Cazelles (2004) was used to investigate the synchrony of the time series under study to determine whether explosivity in one cryptocurrency can lead to explosivity in other cryptocurrencies, namely the possibility that cryptocurrencies co-bubble simultaneously.

**Fig. 3:**
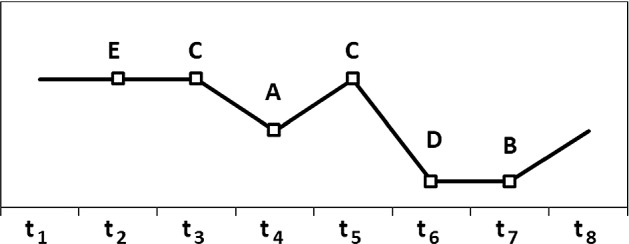
Transformation of a time series into symbols

A time series, $Y = \left\{ {y_{i} } \right\}_{i = 1,2, \ldots , n}$, was first transformed into a sequence of symbols (letters) by comparing each data point $y_{t}$ to its nearest neighbors (the previous and the following record). Thus, $y_{t}$ was identified as a trough point, peak point, increase, decrease, or same in accordance with the following criteria: a trough point if $y_{t} < y_{t - 1} \le y_{t + 1}$ or $y_{t} < y_{t + 1} \le y_{t - 1}$; a peak point if $y_{t - 1} < y_{t + 1} \le y_{t}$ or $y_{t + 1} < y_{t - 1} \le y_{t}$ or $y_{t + 1} \le y_{t - 1} < y_{t}$; an increase point if $y_{t - 1} \le y_{t} < y_{t + 1}$; a decrease point if $y_{t + 1} \le y_{t} < y_{t - 1}$; and, finally, a stability point if $y_{t - 1} = y_{t} = y_{t + 1}$. Symbols A, B, C, D, and E were associated with each of the possible cases (Fig. 3), respectively. Each numerical time series was therefore transformed into a symbolic time series, disregarding any changes in amplitude and mean trend but preserving the fundamental rhythm.

Information theory was then used for quantifying the mutual rhythms of all pairwise combinations of symbolic time series. For any two time series, $Y^{1}$ and $Y^{2}$, the statistical significance of the mutual information was calculated as:

$$ I_{{Y^{1} ,Y^{2} }} = H_{{Y^{1} }} + H_{{Y^{2} }} - H_{{Y^{1} ,Y^{2} }} , $$

where $I_{{Y^{1} ,Y^{2} }}$ represents the mutual information, $H_{Y} = - \mathop \sum \nolimits_{i = 1}^{n} p\left( {y_{i} } \right)\log_{2} \left[ {p\left( {y_{i} } \right)} \right]$ is the entropy of the symbolic series $Y$, $p\left( {y_{i} } \right)$ is the probability that $Y$ could take the value $y_{i}$ and measures the proportion of $y_{i}$ in the time series, and $H_{{Y^{1} ,Y^{2} }} = - \mathop \sum \nolimits_{i = 1}^{n} \mathop \sum \nolimits_{j = 1}^{n} p(y_{i} )p\left( {y_{j} } \right)\log_{2} \left[ {p\left( {y_{i} ,y_{j} } \right)} \right]$ represents their joint entropy. If $Y^{1}$ and $Y^{2}$ are two independent random variables, then $H_{{Y^{1} ,Y^{2} }} = H_{{Y^{1} }} + H_{{Y^{2} }}$ and, therefore, the mutual information $I_{{Y^{1} ,Y^{2} }}$ is zero.

The mutual information was further normalized dividing $I_{{Y^{1} ,Y^{2} }}$ by the sum of the entropy of the two symbolic time series in question, formally:

$$ U_{{Y^{1} ,Y^{2} }} = 2*\frac{{I_{{Y^{1} ,Y^{2} }} }}{{H_{{Y^{1} }} + H_{{Y^{2} }} }}. $$

To assess the statistical significance of the uncertainty coefficient calculated for the cryptocurrencies studied, 500 null mutual information values for any two time series were constructed by a Markov process. If the chance was < 0.05, we rejected the “null” that an observed mutual information value is due to chance. The mutual information values calculated in this way are normalized and can be interpreted as a percentage of synchronization. In particular, the mutual information coefficient allows us to document if time series oscillate at the same rhythm along the respective mean trend (Cazelles 2004).

For the case under study, synchrony analysis is meant to serve the purpose of studying the potential interactions among bubble periods within the cryptocurrency markets investigated. For this reason, we used mutual information over statistical correlations, which instead measure the synchrony between the mean trends of the time series investigated.

Mutual information values and surrogate time series were obtained using an adapted version of the scripts available at https://github.com/people3k/pop-solar-sync (Github 2020).

## Empirical analysis and results

The network degree distribution $P\left( k \right)$, con $k$ = 1, 2, …, represents the fraction of nodes in the graph with a number of direct connections to other nodes (degree) larger than $k$. It can be assumed as a basic measure of heterogeneity of a network.

**Fig. 4:**
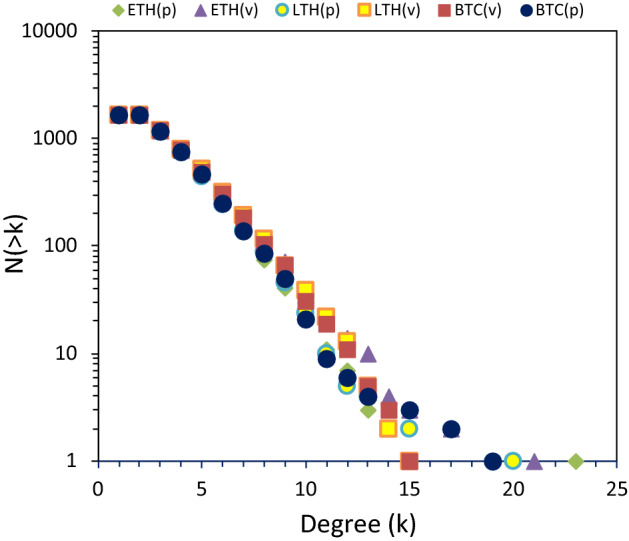
Cumulative degree distributions for the filtered HVGs. Bitcoin (BTC), Ethereum (ETH), and Litecoin (LTC) price (p) and volume (v) series are considered

The cumulative degree distributions for the HVGs associated with the filtered BTC, ETH, and LTH price (p) and volume (v) series are plotted in Fig. 4. They all decay exponentially and are very close to one another. Deviations shown in the tail of the distribution are not significant and are due to the finite nature of the series.

**Fig. 5:**
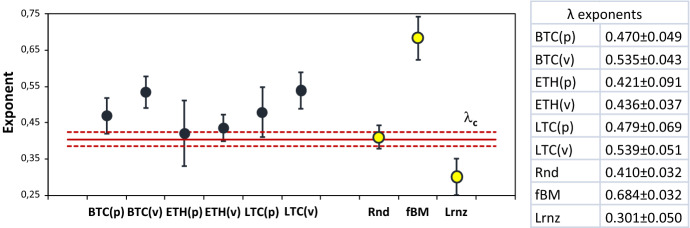
Degree distribution exponents for the HVGs examined and the null models with 95% confidence interval (critical value *λ*_c_ = 0.405 ± 0.020; Rnd = random; fBm = fractional Brownian motion; Lrnz = Lorenz)

The value of the *λ* exponent for the six time series under study is shown in Fig. 5 where bars represent the 95% confidence interval. To better interpret our results, the exponent of the degree distribution for three null models, an uncorrelated stochastic (random) time series (Rnd), a fractional Brownian motion (fBm; the series was generated with Hurst exponent *H* = 0.5), and a series calculated from the Lorenz map (Lrnz; Parker and Chua 1989), was also computed as reference value.

Given $\lambda_{{\text{c}}} = \ln \left( {3/2} \right) = 0.405$, all the $\lambda$ exponents calculated are above such frontier and, therefore, none of the series studied is chaotic. ETH is the only cryptocurrency that exhibits a clear uncorrelated stochastic behavior in both the price and volume series. From $\lambda_{{{\text{BTC}}}} \sim \lambda_{{{\text{LTC}}}} > \lambda_{{\text{c}}}$ emerges that BTC and LTC appear to behave similarly to a correlated stochastic process, instead. For the three virtual currencies studied, the higher value of the $\lambda$ exponent for volumes reveals that traded quantities are more predictable than prices.

A simple comparison between the modularity structure (Table 1) of the HVGs unveils quite the same number of fluctuations over the time horizon considered for the price and volumes series of the three cryptocurrencies investigated.

**Table 1:** Communities and modularity index for the networks

	BTC(p)	BTC(v)	ETH(p)	ETH(v)	LTH(p)	LTH(v)
Modularity (no. commun.)	37	34	35	32	33	32
Modularity (cc)	0.946	0.938	0.945	0.935	0.940	0.932
Modularity normalized	0.972	0.966	0.973	0.965	0.969	0.962

Indeed, for the way the HVG is obtained, each community represents a cycle in the series. Moreover, the high measure (average normalized value = 0.968) for the modularity index proves that cycles in the whole series can be strongly identified.

**Fig. 6:**
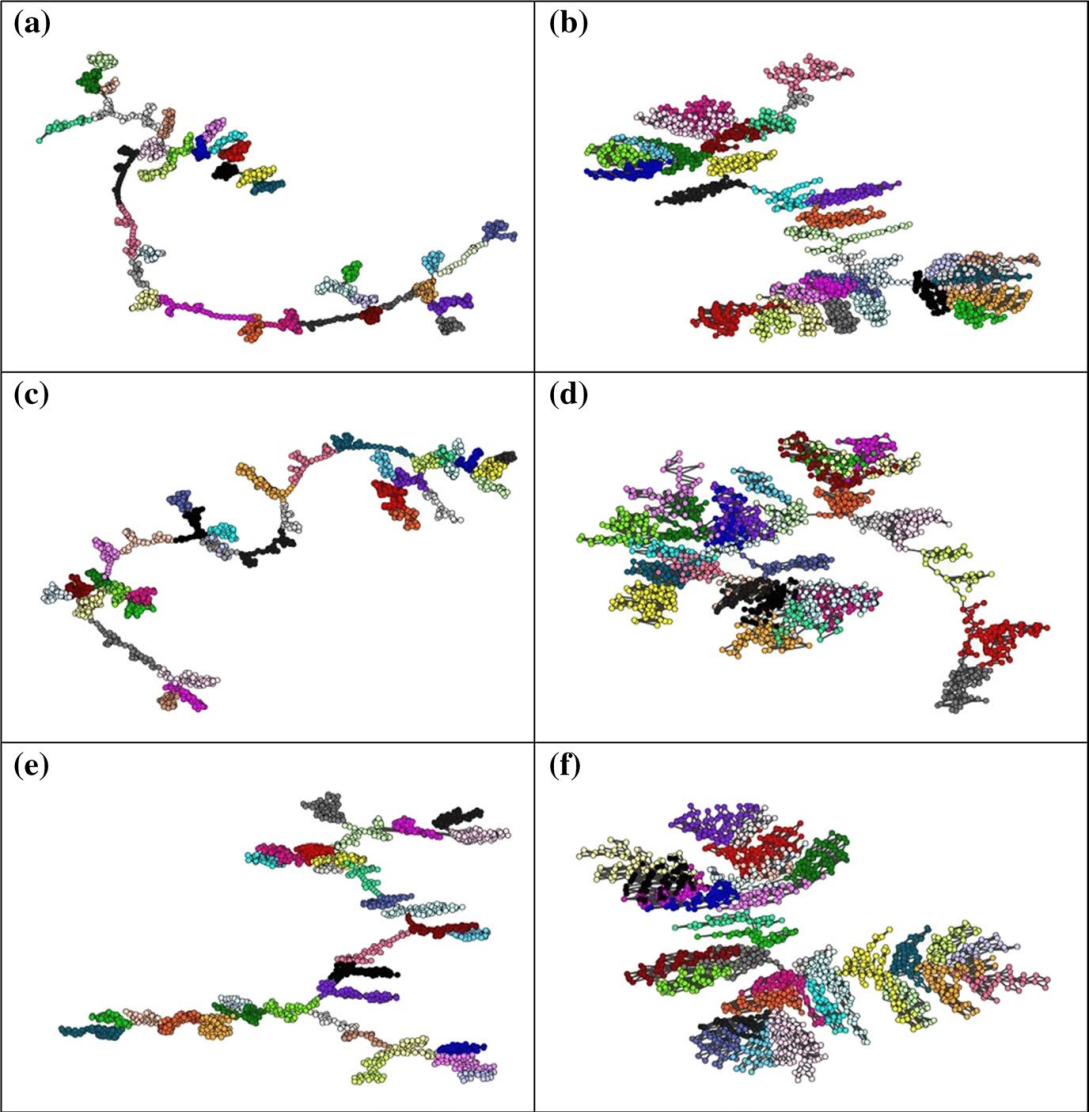
Community structure of the HVGs constructed: **a** BTC(p); **b** BTC(v); **c** ETH(p); **d** ETH(v); **e** LTC(p); **f** LTC(v). Colors (online) are used to mark nodes belonging to the same community

Communities inside the graphs constructed for the virtual currencies investigated are marked with different colors in Fig. 6.

Similarities in the modularity partitions of the HVGs obtained for the three virtual currencies have been also calculated (Table 2) by using a version of Rand index and mutual information (AdjRandIdx, AdjMutInfo) corrected for chance (Hubert and Arabie 1985). These indices are normalized so that the maximum means total agreement between partitions. For comparison, the last two columns show the values calculated using a purely random attribution of community membership (RndRI, RndAMI).

**Table 2:** Similarities in the modularity partitions of the HVGs

		AdjRandIdx	AdjMutInfo	RndRI	RndAMI
BTC(p)	BTC(v)	0.574	0.772	0.001	0.006
BTC(p)	ETH(p)	0.541	0.772	0.001	0.006
BTC(p)	ETH(v)	0.542	0.757	0.001	0.006
BTC(p)	LTH(p)	0.557	0.775	0.001	0.006
BTC(p)	LTH(v)	0.490	0.726	0.001	0.006
BTC(v)	ETH(p)	0.532	0.762	0.000	− 0.002
BTC(v)	ETH(v)	0.615	0.806	0.000	− 0.002
BTC(v)	LTH(p)	0.548	0.772	0.000	− 0.002
BTC(v)	LTH(v)	0.539	0.768	0.000	− 0.002
ETH(p)	ETH(v)	0.553	0.767	0.000	0.001
ETH(p)	LTH(p)	0.537	0.771	0.000	0.001
ETH(p)	LTH(v)	0.479	0.730	0.000	0.001
ETH(v)	LTH(p)	0.530	0.764	0.000	0.001
ETH(v)	LTH(v)	0.593	0.787	0.000	0.001
LTH(p)	LTH(v)	0.519	0.751	0.000	0.004

Results support a strong similarity between the internal (microeconomic) structure of the networks. Not only the HVGs present a roughly equal modularity structure, the internal composition of the communities is alike. To the aim of our analysis, such result proves that price and volume dynamics of the cryptocurrencies were characterized by cyclical patterns of similar wavelength and amplitude over the time period considered.

This result paves the way for the last part of our analysis: the study of synchrony between the virtual currencies investigated.

Time series of price and volumes were compared using the method described in the previous section. The whole time horizon was divided into two subperiods (the vertical line in Fig. 1): before (a) and after (b) February 28, 2018. That date is close to the time of the collapse of price in the Bitcoin market.

Results (Table 3) indicate that the time series investigated display synchrony to a limited extent (marked values are for the pairs that show some higher synchronization). Actually, the relatively low values for the mutual information coefficients obtained do not prove that the time series under investigation are not correlated. They rather document that the three virtual currencies studied, in the time horizon considered, do not show a synchrony such that they might co-bubble simultaneously. Yet, very interestingly, the degree of synchronization between the cryptocurrencies investigated changes before and after a bubble burst occurs. Indeed, with the only exception for LTC, which showed synchrony with the BTC price and volumes series also before the selected date, the value of the uncertainty coefficient increased considerably after the drop in BTC price.

**Table 3:**
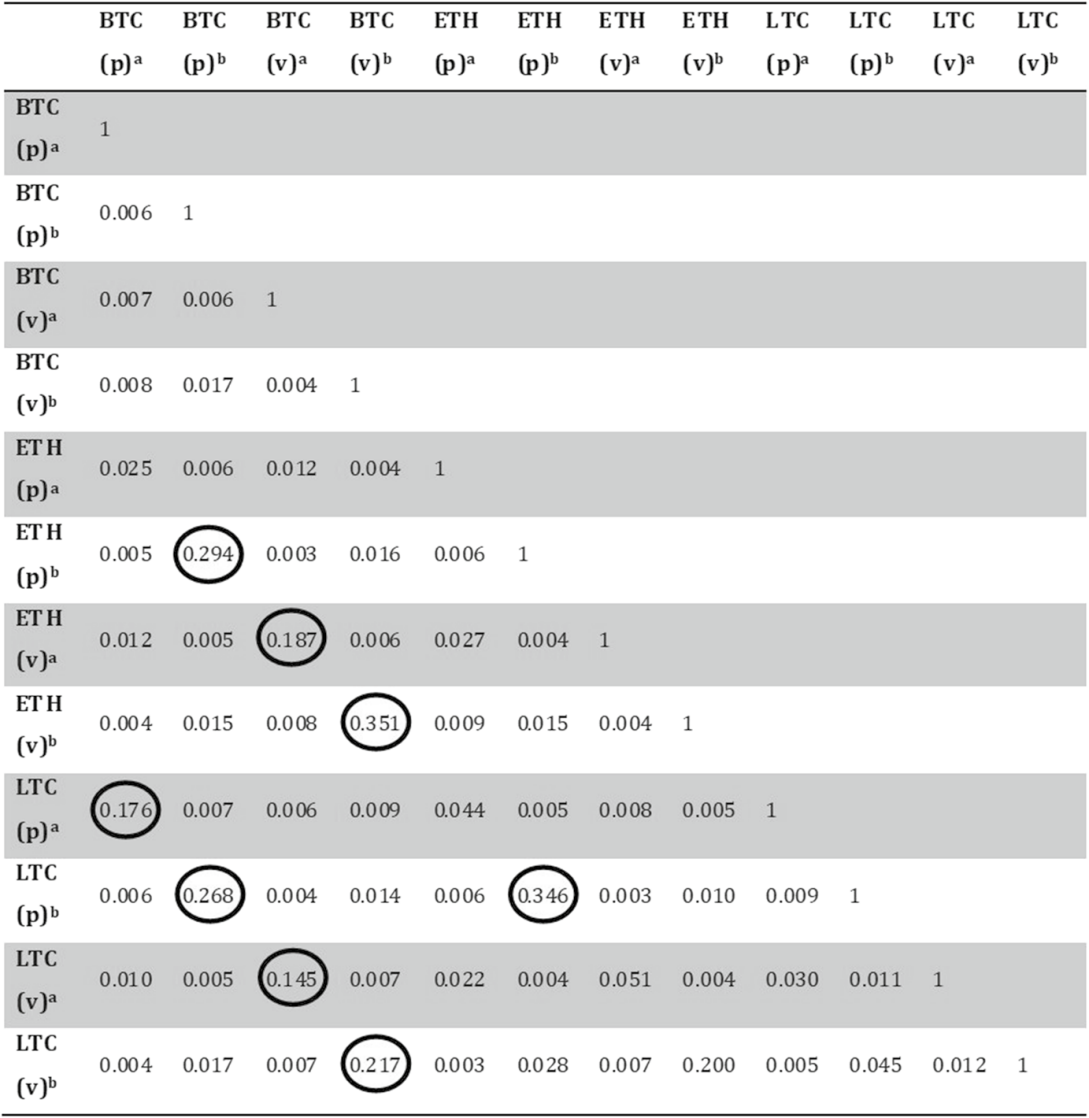
Pairwise normalized (uncertainty coefficient) mutual information values (0 lag) for the price (p) and volume (v) time series

Time horizon split in two periods: before (a) and after (b) 28/02/2018. Higher values, showing higher synchrony, are marked

Although our results do not support the hypothesis of potential interactions among bubble periods, the investigation carried out reveals a higher synchrony in the behavior of the cryptocurrencies investigated after a collapse event.

## Conclusions

In this paper, we provided an extended use of complex network analysis to cryptocurrency market.

Furthermore, we afforded an estimation of the synchrony between the price and volumes series of three virtual currencies, Bitcoin, Ethereum, and Litecoin, based on the procedure described in Freeman et al. (2018) and Cazelles (2004).

Price and volume time series were first mapped into the associated horizontal visibility graphs to reveal the complex structure of the virtual currencies studied. Afterwards, synchrony analysis was carried out to unveil possible common patterns of upswing and collapse in the dynamics of price and volumes for the virtual currencies investigated.

Summing up the main outcome, we can conclude that BTC, LTC, and ETH exhibit different dynamics: correlated stochastic for BTC and LTC, and a clear uncorrelated stochastic (random) behavior for ETH. Moreover, volume dynamics resulted more predictable than prices. A strong similarity was found between the currencies investigated in terms of their community structure, instead. Such result proves that price and volume dynamics of the three virtual currencies were characterized by cyclical patterns of similar wavelength and amplitude over the time period considered. Yet, as the synchrony analysis revealed, such similarity did not translate into the possibility for the cryptocurrencies to co-bubble simultaneously. Indeed, with the only exception represented by the dynamics shown by the Litecoin market, the values of the uncertainty coefficient calculated for the other two cryptocurrencies changed considerably before and after the time of the collapse of price in the Bitcoin market. Although our results do not support the hypothesis of potential interactions among bubble periods, the investigation carried out revealed a higher synchrony in the behavior of the cryptocurrencies investigated after a collapse event.
